# Diplodia Tip Blight on Its Way to the North: Drivers of Disease Emergence in Northern Europe

**DOI:** 10.3389/fpls.2018.01818

**Published:** 2019-01-09

**Authors:** Laura Brodde, Kalev Adamson, J. Julio Camarero, Carles Castaño, Rein Drenkhan, Asko Lehtijärvi, Nicola Luchi, Duccio Migliorini, Ángela Sánchez-Miranda, Jan Stenlid, Şule Özdağ, Jonàs Oliva

**Affiliations:** ^1^ Department of Forest Mycology and Plant Pathology, Swedish University of Agricultural Sciences, Uppsala, Sweden; ^2^ Pyrenean Institute of Ecology (IPE-CSIC), Zaragoza, Spain; ^3^ Sustainable Forest Management Research Institute, University of Valladolid, Palencia, Spain; ^4^ Institute of Forestry and Rural Engineering, Estonian University of Life Sciences, Tartu, Estonia; ^5^ Faculty of Forestry, Bursa Technical University, Bursa, Turkey; ^6^ Institute for Sustainable Plant Protection, Italian National Research Council, Firenze, Italy; ^7^ Isparta University of Applied Sciences, Isparta, Turkey; ^8^ Department of Crop and Forest Sciences–Agrotecnio Center CERCA, University of Lleida, Lleida, Spain

**Keywords:** carbon isotopes, dendroecology, water-use efficiency, latewood, earlywood, vascular wilt pathogen

## Abstract

Disease emergence in northern and boreal forests has been mostly due to tree-pathogen encounters lacking a co-evolutionary past. However, outbreaks involving novel interactions of the host or the pathogen with the environment have been less well documented. Following an increase of records in Northern Europe, the first large outbreak of *Diplodia sapinea* on *Pinus sylvestris* was discovered in Sweden in 2016. By reconstructing the development of the epidemic, we found that the attacks started approx. 10 years back from several isolated trees in the stand and ended up affecting almost 90% of the trees in 2016. Limited damage was observed in other plantations in the surroundings of the affected stand, pointing to a new introduced pathogen as the cause of the outbreak. Nevertheless, no genetic differences based on SSR markers were found between isolates of the outbreak area and other Swedish isolates predating the outbreak or from other populations in Europe and Asia Minor. On a temporal scale, we saw that warm May and June temperatures were associated with higher damage and low tree growth, while cold and rainy conditions seemed to favor growth and deter disease. At a spatial scale, we saw that spread occurred predominantly in the SW aspect-area of the stand. Within that area and based on tree-ring and isotope (δ^13^C) analyses, we saw that disease occurred on trees that over the years had shown a lower water-use efficiency (WUE). Spore traps showed that highly infected trees were those producing the largest amount of inoculum. *D. sapinea* impaired latewood growth and reduced C reserves in needles and branches. *D. sapinea* attacks can cause serious economic damage by killing new shoots, disrupting the crown, and affecting the quality of stems. Our results show that *D. sapinea* has no limitations in becoming a serious pathogen in Northern Europe. Management should focus on reducing inoculum, especially since climate change may bring more favorable conditions for this pathogen. Seedlings for planting should be carefully inspected as *D. sapinea* may be present in a latent stage in asymptomatic tissues.

## Introduction

Globalization and climate change are driving forest pathogen invasions and disease emergence worldwide (Stenlid and Oliva, 2016). While reports on human-mediated movements of pathogens continue to cumulate (Liebhold et al., 2012; Jung et al., 2016; Redondo et al., 2018b), few cases where new outbreaks are appearing in connection with climate change have been documented (La Porta et al., 2008). Climate may limit disease by means of different, and often interacting, mechanisms, making climate-change driven outbreaks difficult to understand and predict (Sturrock et al., 2011). One possibility is that climate is limiting the distribution of the pathogen. A good example of such mechanism is the cold winters presumably limiting the northwards expansion of *Phytophthora x alni* (Brasier and S.A. Kirk) Husson, Ioos and Marçais on alder (*Alnus glutinosa* (L.) Gaertn.) (Redondo et al., 2015). The second possibility is that the pathogen is present, but climate is limiting the capacity of the pathogen to cause disease for instance by reducing its capacity to build inoculum. A good example of such mechanism was seen for *Dothistroma septosporum* (Dorogin) M. Morelet in British Columbia where large damages occurred on lodgepole pine forests (*Pinus contorta* Douglas ex Loudon) along with an increase of summer rainfall during the previous decades (Woods et al., 2005). The third and less well-documented possibility is when climate is limiting host susceptibility. In that case, outbreak etiology is complex and may involve maladaptive phenotypes (Stenlid and Oliva, 2016), changes in stress regimes (Hanso and Drenkhan, 2013; Oliva et al., 2014), changes in phenology, or the combination of both (Françoise et al., 2015).

Predicting new forest disease outbreaks also needs to consider their geographic location. Climate change for instance has been suggested to increase forest damage in northern latitudes (Sturrock et al., 2011). However, still little is known about the particular processes behind disease emergence under these conditions. For instance, short growing seasons may make trees growing in northern locations more sensitive to needle loss than their southern counterparts (Oliva et al., 2016; Stenlid and Oliva, 2016). Also, a longer daytime during summer may also extend the periods when trees photosynthesize and expose themselves to water stress. Nevertheless, more cases are needed to depict general patterns. New and, so far, innocuous pathogen encounters are increasingly reported in Northern areas (Millberg et al., 2016; Redondo et al., 2018a,b), and we lack tools to predict their potential impact.

The case of *Diplodia sapinea* (Fr.) Fuckel (syn. *Diplodia pinea* (Desm.) Kickx., *Sphaeropsis sapinea* (Fr.: Fr.) Dyko and Sutton) in Sweden represents a good example of emergence in Northern forests. The pathogen was reported for the first time in 2013 and was regarded innocuous as no associated damages were observed (Oliva et al., 2013). However, 3 years after the first observation, *D. sapinea* was found associated with an unprecedented outbreak on Scots pine (*Pinus sylvestris* L.). In August 2016, stand-level damages affected a circa (ca.) 15 ha plantation north from Stockholm, where hundreds of 20-year-old Scots pine trees appeared severely damaged. Well-developed trees had lost completely all current year’s shoots, and some of them were dead. The incidence was high, with the majority of trees affected, in most cases having lost their main leader shoot. A closer look in the stems revealed that most trees were either bifurcated or displayed bushy crowns, suggesting that they had probably suffered *D. sapinea* attacks in the past.

The outbreak in Sweden not only represented a qualitative change to the previous behavior of this pathogen in Northern Europe but also at a global scale. *Diplodia sapinea* had been historically reported in Southern and Central Europe causing shoot dieback, canker, blue stain, and root disease on pines (Fabre et al., 2011; Luchi et al., 2014). Mirroring the situation in Europe, in the Southern Hemisphere, *D. sapinea* had caused outbreaks in areas with mild climates, such as South Africa and New Zealand, where mostly non-native pine plantations have been established (Burgess et al., 2004). The pathogen had also been detected in relatively cold areas, but recent detections in Estonia (Hanso and Drenkhan, 2009), Finland (Müller et al., 2018) and north-western Russia (Adamson et al., 2015) seemed to suggest an ongoing range expansion to the north. Following that pattern, pycnidia of *D. sapinea* were observed for the first time in 2013 (59°N) on a Scots pine cone in Sweden (Oliva et al., 2013). Further samplings done in that same year found pycnidia in cones all over Southern Sweden, in areas such as Uppsala, Gothenburg, Malmö, and Visby, on both non-native *Pinus nigra* Arnold and Scots pine. In spring 2014, a small group of trees displaying shoot blight damages from previous year (2013) were found north from Gothenburg, though as in previous cases, symptoms corresponded to isolated branches or trees.

In this research, we aimed at understanding the causes of the *D. sapinea* outbreak in Sweden, as a way to improve our capacity to predict disease emergence in northern conifer forests. We did so by testing five explicit hypotheses on disease emergence. The first hypothesis concerned the origin of the pathogen. Until 2016, the steady increase of reports in Northern Europe fitted well in the picture of a pathogen slowly expanding its range. However, the isolated nature of the outbreak and the high severity of damage over several hectares raised the question of whether the observed damages could be the result of introduction of a new aggressive strain or a cryptic species of the pathogen (Hypothesis 1). We tested hypothesis 1, by studying the population structure of the pathogen in the outbreak area in comparison with that of isolates previously discovered in Sweden in areas with no apparent damage and with isolates of other parts in Europe, such as Estonia, Spain, and Italy, as well as from Turkey.

A regional survey of other attacks was also attempted in order to gain insights on the origin of the outbreak. Confirming an isolated nature of the outbreak (Hypothesis 2) could support the idea that infected planting stock was perhaps used in that particular stand. In forest nurseries in Wisconsin (USA), up to 27% of red pine seedlings were found to carry latent infections (Stanosz et al., 1997), and we know that in the 1950s, *D. sapinea* was present in Swedish forest nurseries (Molin et al., 1961). Thus, in order to test hypothesis 2, we surveyed the surroundings of the outbreak area for stands also affected by *D. sapinea*.

The role of weather on disease development was also explored. *D. sapinea* enters the host through stomata of elongating needles at expanding shoots and injured tissue (Brookhouser and Peterson, 1971). *D. sapinea* remains in a latent stage in asymptomatic trees (Smith et al., 1996), while disease is often induced by stress factors such as drought (Bachi and Peterson, 1985; Stanosz et al., 2001), hail (Zwolinski et al., 1990), or mechanical wounding (Chou, 1987). At regional scale, severity of damages has been associated with higher temperatures in Italy (Bosso et al., 2017) and France (Fabre et al., 2011); however, the role of weather in northern latitudes is unknown. We hypothesized that weather conditions played a role on the development of the *D. sapinea* outbreak (Hypothesis 3). In order to test hypothesis 3, we reconstructed the *D. sapinea* epidemic in the stand by inferring the time when trees had lost the leader shoot. We modeled the development of the epidemic and correlated departures from the expected disease progression curve with tree growth and monthly weather conditions.

Damages were not homogeneous across the stand, pointing to disease development being modulated by spatial features. Across the outbreak, there was also variation in terms of damage among trees. Within the same plot, highly damaged trees were found nearby asymptomatic trees, also pointing to the role of some tree features in the development of the outbreak. Disease in northern latitudes has been sometimes associated with maladaptive phenotypes (Stenlid and Oliva, 2016), such as pine trees planted in fertile sites that would normally correspond to Norway spruce (*Picea abies* (L.) Karst.) (Witzell and Karlman, 2000). Given the link between drought and Diplodia shoot blight, we hypothesized that Scots pine phenotypes with lower water use efficiency (WUE hereafter) would be more susceptible to *D. sapinea* (Hypothesis 4). In order to test hypothesis 4, we reconstructed radial growth (earlywood and latewood widths), quantified non-structural carbohydrate (NSC hereafter) concentrations in several tissues (branch, needles), and measured wood C isotope discrimination (δ^13^C) of trees severely attacked by *D. sapinea* in comparison with asymptomatic trees growing nearby.

Understanding the spatiotemporal development of the outbreak was also attempted by focusing on the spread of the pathogen. *Diplodia sapinea* has large spores that are released from pycnidia by raindrop splashes (Brookhouser and Peterson, 1971; Swart et al., 1987), thus we expected that *D. sapinea* would disperse in short distances in the studied stand (Hypothesis 5). We tested hypothesis 5 by comparing spore captures underneath diseased trees along a range of severity of symptoms, and between wet and dry periods. We also explored the use of qPCR instead of time-consuming spore counting measures for future monitoring purposes.

## Materials and Methods

### Population Structure Studies

The affected stand was located next to a highway (E4), northwest from the Stockholm Arlanda Airport (59°40′51.2″N, 17°52′20.8″E), and it corresponded to a ca.15 ha Scots pine plantation established in 2000 around some older Norway spruce forest patches.

**Figure 1:**
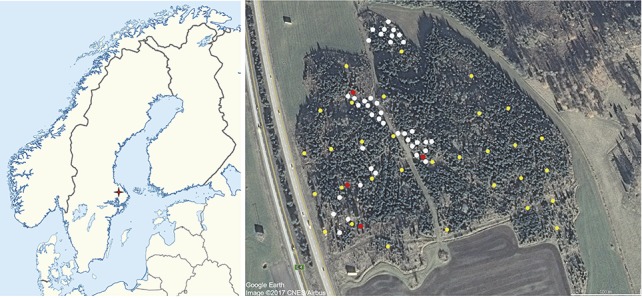
Location of 40 trees used for isolation and spore trapping (white), 31 plots used for damage assessment and dendrochronology (yellow), and location of the four plots where the 24 trees used for physiological measurements were located within the affected stand (red). Image on the left modified from NordNordWest - own work, using World Data Base II data, CC BY-SA 3.0. Image on the right modified from Google Earth Image © 2017 CNES/Airbus.

In order to confirm the identity of the pathogen causing the outbreak, three attacked shoots per tree from 40 trees were collected (Figure 1). Five wood pieces were taken from the margins of necrotic tissues of each shoot samples and were placed in a plate containing 2% malt extract agar. After a two-day incubation at 21°C, fungal colonies were randomly picked up from three of the wood pieces, and they were transferred to a new plate. After two weeks, one of the three isolates was chosen by its morphological similarity with the rest and recultured a second time for DNA extraction. After 5 days at 21°C, two pieces (~0.5 cm^2^) of mycelium were harvested and stored at −20°C. DNA from all 40 isolates was extracted with a NaOH/Tris-HCl-based method described in Wang et al. (1993).

We searched for signs of genetic differentiation between *D. sapinea* isolates in the outbreak area and isolates from Sweden, Europe, and Asia Minor (Turkey). *D. sapinea* population from Sweden consisted of 92 isolates. The collection was obtained in an isolation campaign carried out in 2013, when single-spore isolates were obtained from infected pine cones from five different locations: Visby (four *P. nigra* cones), Lomma (four *P. nigra* cones), Gothenburg (four *P. mugo* Turra cones), Uppsala (one *P. sylvestris* cone), and Fjällnora (one *P. sylvestris* cone). Single-spore isolates were taken by collecting pycnidia from cones and dissolving them in water and plating them in water agar. Individual germlings were retransferred to malt extract agar. For comparison with the rest of Europe and Asia Minor, a total of 65 isolates from Estonia (two locations, 11 isolates), Spain (five locations, 23 isolates), Italy (two locations, 15 isolates), and Turkey (two locations, 16 isolates) were included. The isolates in Spain, Italy, and Turkey were obtained in a similar scheme as Swedish ones from 2013, that is, single-spore isolates from cones. Estonian isolates were collected by direct isolation from symptomatic shoots and cones.

Isolates used for population structure were confirmed to be *D. sapinea* by being positive based on the specific PCR essay from Smith and Stanosz (2006). No major morphological differences were observed among the isolates of the outbreak area; therefore, a subsample of seven out of 40 was sequenced. Sequencing targeted the internal transcribed spacer (ITS) 1 and 2 and the 5.8S ribosomal RNA gene with the ITS1f and ITS4 primers (Gardes and Bruns, 1993). The purified PCR products were sequenced by Macrogen (Macrogen Inc., Seoul, Korea), edited in SeqMan pro (DNAStar, Inc., Madison, WI, USA; version 12.0.0), and blasted in GenBank (Genbank accession numbers: MK120100 - MK120106).

We searched for genetic differentiation among populations by looking for allelic variation in 10 simple sequence repeat (SSR) markers (Burgess et al., 2001; Bihon et al., 2011). The original panel of 16 markers was shortlisted to the ones that showed any variability. SSRs were amplified following Burgess et al. (2001). Amplification products were diluted 10- to 200-fold and send to Uppsala Genome Centre for fragment size analysis. SSR alleles were discriminated with the GeneMarker software (Softgenetics, State College, PA). Population structure analyses were carried out with the package POPPR (Kamvar et al., 2014) for R version 3.0.3 (The R Foundation for Statistical Computing). Combining the data of the 10 SSR markers, a multilocus genotype (MLG) was determined for each isolate. Swedish populations collected in 2013 and 2016 and the rest of European populations were compared in terms of rarefied numbers of MLGs and Simpson’s index of diversity. Geographical differentiation among European countries was tested by means of a MANOVA analysis, calculated based on D-Jost distance, and visualized with a minimum spanning network. D-Jost values and statistical significance were calculated by bootstrapping with and without clone correction in the R package DEMEtics v.0.7-8.

In order to further support species identification, a sporulation test was conducted for two isolates from the outbreak area belonging to two different haplotypes. Isolates were cultivated on autoclaved pine needle extract (*P. sylvestris* needles ground on liquid nitrogen, 2% Agar) (Luchi et al., 2007). Cultures were kept under constant fluorescent light at 28^°^C. Pycnidia formed after one week on the surface of the agar and pine needle debris at the ground of the culture. After two weeks, five mature pycnidia per isolate were harvested. Conidial length and width, color, septation, and wall texture of 62 spores were measured from digital images recorded with a Leica DFC285 camera (Leica Microsystems, Switzerland) attached to a microscope (Axioplan, Carl Zeiss AG, Germany) and processed with the NIH imageJ software (version 1.52b, http://rsb.info.nih.gov/ij/). Morphological and morphotype identification followed the observations and identification key of Palmer et al. (1987) and Phillips et al. (2013).

In October 2016, thirteen Scots pine stands situated in a radius of 5 km from the outbreak area were surveyed. A second survey was undertaken in May 2017 including 28 Scots stands with a similar age as the plantation of the initial outbreak in a radius of 30 km. Shoots displaying symptoms were examined for characteristic pycnidia and microscopically screened for spores of *D. sapinea*, but no isolations were undertaken. Isolates from surveys around the infected area in 2016 were not included in the population structure analysis.

### Tree Measures and Assessment of Damage

In October 2016, a systematic tree assessment was carried out across the outbreak area. A total of 264 pines within 31 plots were measured (Figure 1). Plots were circular and had a 2 m radius (12.6 m^2^). In each plot, stem girth, height, and infection level of all trees with a girth larger than 3 cm at breast height were measured. Signs of bifurcation in the stem as well as the infection of the leader shoot were recorded. Stem girth was measured at breast height. The infection level per tree was estimated as the percentage of infected shoots proportional to all shoots from the upper third of the living crown. Signs of previous bifurcations were present all over the stand, thus timing of former attacks was inferred by recording the number of internodes (years) after the bifurcation, that is, after the leader shoot was lost. Additionally, a measure of exposure of the crown to wind and rain was taken in a scale from 0 to 4; according to which, a value of 4 indicated that the tree had a fully exposed crown, while a value of 0 indicated that the tree was completely surrounded by other trees. We extracted two increment wood cores from each tree at breast height using a Pressler increment borer. Wood cores were air-dried and their surface was carefully sanded until tree-ring boundaries were clearly visible. Then, tree rings were visually cross-dated and earlywood and latewood widths were separately measured with precision of 0.001 mm using a binocular microscope and the LINTAB package (Rinntech, Heidelberg, Germany). Earlywood and latewood widths were visually distinguished by experienced dendrochronologists based on the change in wood color and tracheid transversal dimensions. Tree-ring widths were transformed into basal area increments (BAI), assuming a circular shape of the stem. The COFECHA program (Holmes, 1983) was used to evaluate the visual cross-dating of tree-ring series.

### Physiological Comparison Between *D. sapinea* Defoliated and Non-defoliated Trees

In February 2017, six pairs of defoliated and non-defoliated trees were selected in four different plots within the outbreak (*n* = 24 trees). In each plot, defoliated/non-defoliated tree pairs were stratified by size, so two big-diameter trees, two medium-sized, and two small trees were measured. Trees were chosen to be as close as possible (max. distance of 5 m). Trees were felled and discs were cut from the base and below the living crown. Tree growth was obtained from wood discs as done from increment wood cores. To couple growth measures with water-use efficiency, we compared carbon isotope ratios (^13^C/^12^C, δ^13^C) in wood formed in the years 2012, 2013, 2014, and 2016 between healthy and infected trees yielding a total of 96 wood samples. The years 2012 and 2014 corresponded to years with colder-than-normal spring temperatures, while spring temperatures in 2013 and 2016 were warmer-than-normal. The wood samples for δ^13^C analyses were dried in the oven at 70°C for 48 h, then whole annual tree rings were separated using scalpels, and the resulting wood samples were ground to a fine powder. Wood aliquots (0.001 g) were weighed on a balance (AX205 Mettler Toledo, OH, USA) into tin foil capsules and combusted using a Flash EA-1112 elemental analyzer interfaced with a C isotope ratio mass spectrometer (Thermo Fisher Scientific Inc., MA, USA). Isotope analyses were conducted at the UC Davis Stable Isotope Facility (Davis, USA). Stable isotope ratios were expressed relative to Vienna Pee Dee Belemnite (VPDB). The standard deviation was better than 0.1‰.

Recently formed (one-year old) needles, branch sapwood, and root samples were also taken in order to assess whether NSC concentrations differed between damaged and undamaged trees. From damaged trees, samples were taken from needles and branches proximal to the damaged area, so comparison with the effects of *D. sapinea* defoliation with distal tissues, such as roots, could be possible. Samples were transported to the laboratory in a portable cooler, where they were frozen and stored at −20°C until freeze-dried. Samples were weighed and milled to a fine and homogeneous powder using a ball mill (Retsch Mixer MM301, Leeds, UK). Soluble sugars were extracted with 80% (v/v) ethanol, and their concentration was colorimetrically determined using the phenol-sulfuric method (Buysse and Merckx, 1993). Starch and complex sugars remaining after ethanol extraction were enzymatically digested with an enzyme mixture containing amyloglucosidase to reduce glucose as described in Palacio et al. (2007). NSC measured after ethanol extractions were regarded as soluble sugars, whereas carbohydrates measured after enzymatic digestion were considered to be mostly starch. The NSC concentration was calculated as the sum of soluble sugars and starch concentration.

### Quantification of Spore Production Under Infected Trees

Forty spore traps were placed directly underneath forty trees with varying levels of damage (Figure 1). Spore traps had a height of about 50 cm and consisted of one horizontally fixed filter paper (Munktell, Ahlstrom; Ø90 mm) and one microscopy slide covered with two stripes of tape coated with permanent adhesive on both sides (Scotch® Double Sided Office Tape). The first set of traps was placed in 2016, from 30th of September to the 10th of October during a period with little rain (total precipitation 3.6 mm, 3 days of rain, average temperature 8.0°C); the second set was placed from the 21st of October to the 31st, during a rainy period (total precipitation 24.4 mm, 7 days of rain, average temperature 5.4°C). For spore counting, microscopy slides were divided into 22 rectangles of 0.5 cm × 1.3 cm each. Half of those 22 rectangles were screened under the microscope, where *D. sapinea* spores were distinguished by morphological characteristics (Cheng-guo et al., 1985). In order to evaluate the possibilities of monitoring *D. sapinea* by qPCR, we compared the number of spores counted in the microscope slide with the quantity obtained in terms of copy numbers from qPCR. For that, DNA was extracted from filter papers, by placing the entire filter in a 50-ml falcon tube under sterile conditions. After addition of 20 ml SDS buffer, filters were incubated for 90 min at 65°C. Next, the filter paper was removed and 20 ml of isopropanol was added and incubated overnight. On the following day, the sample was centrifuged at 7000 rpm for 10 min and the supernatant removed. From there and on, DNA extraction was continued with the NucleoSpin® Macherey-Nagel Soil Kit, following manufacturer’s instructions. qPCR was done following the TaqMan^TM^ setup designed by Luchi et al. (2005). Each 20 μl reaction consisted of a final concentration of 1× SsoAdvanced^TM^ Universal Probes Supermix (BioRad), 250 nM of each primer, 200 nM probe, and 1 μl DNA extract/1 μl sterile water as non-template control. The qPCR program consisted of 2 min at 95°C, followed by 40 cycles of 10 s at 95°C and 15 s at 60°C. Copy numbers were obtained by averaging three technical replicates of each sample.

### Data Analysis

The spatial association between disease and growth was examined by correlating the number of attacked trees and the relative X and Y coordinates of the plots (*n* = 31). A composite variable representing a gradient from SW to NE was calculated as the product of the coordinates. The development of the disease in each plot in time was modeled as linear function (*n* = 9 years), and the significance of that correlation was calculated. The correlation was used as a measure of disease increment and was also regressed against the SW-NE location of the plot. When modeling tree features associated with damage, we ran a stepwise selection to reduce the number of variables, always including the plot as a random factor.

We performed an epidemiological analysis in relation to tree growth and weather. We reconstructed the epidemic by pooling the number of records of attacks among all trees in the stand (*n* = 264). Development with time was modeled with a linear function (*n* = 9 years). BAI measures were integrated for all trees in the stand (*n* = 264) and were also regressed against time with a linear function (*n* = 9 years). Departures from a linear increase in both the epidemiological and the growth model were transformed into studentized residuals, which were then correlated with month’s daily average temperature and precipitation sum for the period 2007 to 2016. Weather data for the outbreak stand were obtained by the interpolation tool provided by the Swedish Meteorological Institute and Hydrological Institute and available on the web.^1^

Earlywood and latewood widths were compared between pairs of trees defoliated by *D. sapinea* (*n* = 12) and non-defoliated trees (*n* = 12) separately for every year from 2007 to 2016. δ^13^C comparisons were done similarly, but in that case all years were considered a factor in the analysis and the particular tree was included as a random factor in a mixed model. The degrees of freedom were adjusted by a Kenward-Roger approximation. Comparisons of NSCs and their different fractions between defoliated (*n* = 12) and non-defoliated trees (*n* = 12) were done by an ANOVA analysis including the plot as a blocking factor in the model.

The association between spore captures and tree features was done by regression (*n* = 40 and 39 traps in wet period and dry period respectively). Inclusion of variables in the model was done by stepwise selection. The association between spore captures and location was calculated by correlating the number of spores with the coordinates of each particular spore trap obtained by GPS *in situ*. Correlation between qPCR values and spore counts was done on data of 77 traps as some paper filters were lost during the rainy week. We tested whether the correlation between gene copies number (qPCR of filter paper trap) and spores (sticky traps) was different during the rainy and the dry week by including “week” as a factor in the model, both in the intercept and the slope “week × spore number.” All analyses were carried out in Minitab® 18.1 for Windows.

## Results

### Genetic Background of the Isolates in the Outbreak Area

Diplodia-like colonies represented a 76% of the isolates obtained from symptomatic tissues in the outbreak area. Isolates showed first a fast-growing white mycelia which turned to gray/black as cultures became old. Isolates obtained from the outbreak area corresponded to *D. sapinea* with a 100 % match to the sequence of *Sphaeropsis sapinea* 18S ribosomal RNA gene (GenBank: JF440618.1). Those, and the rest of isolates from Europe, included in the population structure analysis were positive for *D. sapinea* based on specific primers.

**Figure 2:**
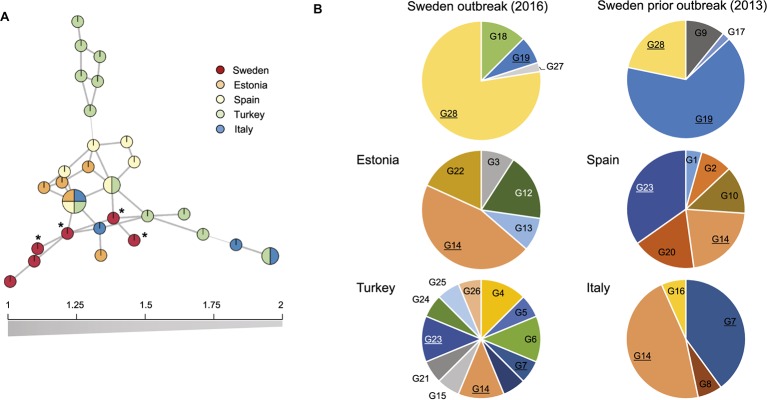
Minimum spanning network among *D. sapinea* haplotypes in Europe **(A)**. Haplotypes from the outbreak area are marked with an (*). Pie charts with relative proportion of haplotypes among populations **(B)**. Underlined haplotypes are found in more than one location.

Based on 10 SSR markers, the European population of *D. sapinea* was highly clonal, as only 28 MLG were found among 197 isolates. Rarefied numbers of MLG showed between 3 and 5 MLG (per 10 isolates) among all studied countries with the exception of Turkey, whose isolates displayed a distinct and much higher diversity than the other areas (9 MLG per 10 isolates) (Table 1). We found signs of geographic differentiation between European populations, accounting for 24% of the genetic variance (*p* < 0.001) (Figure 1). The Estonian population was the least differentiated from the rest (average D-distance of 0.05), while Swedish populations was the most dissimilar from the others (average distance 0.15), in particular from southern Spanish and Turkish populations (Table 1). Minimum spanning network showed a cluster of Turkish isolates highly differentiated from the rest (Figure 1). When using clone correction, significant differentiation was only found between Sweden and Turkey (D-Jost = 0.14, *p* = 0.003). No differentiation was found among Swedish populations (Table 1). The isolates of the outbreak were not genetically different from isolates previously obtained in Sweden (Figure 2), although they appeared to be slightly less diverse. The most abundant MLG in the outbreak, arbitrarily named G28, was also relatively abundant in 2013. That same haplotype was not found anywhere else in Europe. Spore size of G28 was similar to that of the haplotype G19, the most abundant in Sweden before the outbreak (length: 40.0 vs. 40.9 μm, *p* = 0.08; 15.4 vs. 15.5 μm, *p* = 0.85, respectively). In both cases, conidia produced *in vitro* were non-septated and showed a rough wall texture. Spore size, shape, and number of septa matched those of morphotype A.

**Table 1:** Population structure among *Diplodia sapinea* isolates from five different locations in Sweden collected in 2013 and from the location of the outbreak, and genetic distance among Sweden’s *D. sapinea* population and other four populations in Europe.

Population	*n*	MLG	eMLG	Simpson’s index corrected	Jost’s D genetic distance				
**Sweden**					**Arlanda**	**Fjällnora**	**Gothenburg**	**Gula Stigen**	**Lomma**
Arlanda, 2016	40	4	2.62	0.387					
Fjällnora, 2013	7	2	2	0.286	−0.05				
Gothenburg, 2013	9	2	2	0.223	**0.03**	0.00			
Gula Stigen, 2013	31	2	1.93	0.322	0.02	−0.01	−0.04		
Lomma, 2013	20	3	2.98	0.679	0.00	−0.02	0.00	−0.02	
Visby, 2013	25	4	2.99	0.510	**0.04**	0.02	−0.01	-0.03	−0.02

**Europe**					**Sweden**	**Estonia**	**Spain**	**Turkey**	
Sweden	132	6	3.19	0.620					
Estonia	11	5	5.00	0.782	**0.12**				
Spain	23	6	5.01	0.810	**0.14**	0.01			
Turkey	16	12	9.17	0.966	**0.19**	**0.07**	**0.07**		
Italy	15	4	3.47	0.657	**0.16**	0.02	**0.06**	**0.07**	

Data based on 10 SSR loci. The number of isolates (n), multilocus genotypes (MLG), multilocus genotypes rarefied to 10 isolates (eMLG), and Simpson index corrected for different sample size are shown. Jost’s D genetic distance calculated without clone correction. Jost’s D-values significantly different from 0 are shown in boldface.

### Spatiotemporal Spread of the Epidemic

**Figure 3:**
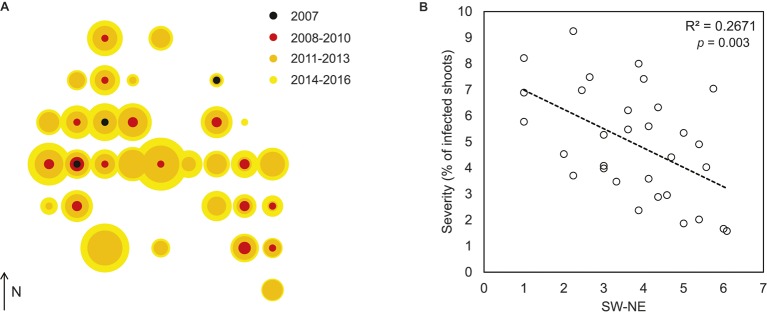
Spatial and temporal spread of *D. sapinea* within the affected Scots pine stand **(A)** and association between severity in 2016 and location (*n* = 31 stands) **(B)**. SW-NE was calculated as the product of the relative X and Y coordinates of the sampling plots; higher values indicate NE locations, while low values indicate SW locations. Both severity and SW-NE location are plotted following square-root transformation.

Symptomatic trees with similar symptoms were found in 7 of 13 stands in the vicinity of the outbreak area (<5 km). Further away (<30 km), incidence was much lower, with 3 of 28 stands with signs of *D. sapinea*. In any of the surveyed stands, damage severity resembled the one observed in the outbreak area. Across the outbreak, 85% of the trees were infected, while 53 % showed damage on the leader shoot. Among attacked trees, almost a third of the shoots in the upper third of the crown were affected (28%). There was a clear spatial pattern across the stand, where severity appeared to be higher in the southwest area of the stand (Figure 3). Based on the number of previous loses in the leader shoot, we reconstructed the epidemic back to 2007. Three plots situated in the north/north-western area of the stand had the oldest attacks (Figure 3). From 2007 onwards, we observed a significant (*p* < 0.05) increase of the number of attacks over the years in ca. half of the plots (48%). The largest increments were observed in the southwest area of the stand (*R* ^2^ = 0.13, *p =* 0.043). At plot level, no association between disease progression or damage and diameter, height, exposure, or tree growth was found. At tree level, the percentage of dead shoots correlated with both the number of previous attacks (*p* < 0.001) and tree height (*p* = 0.006).

### Weather Conditions Associated With Tree Growth and Disease Increase

**Figure 4:**
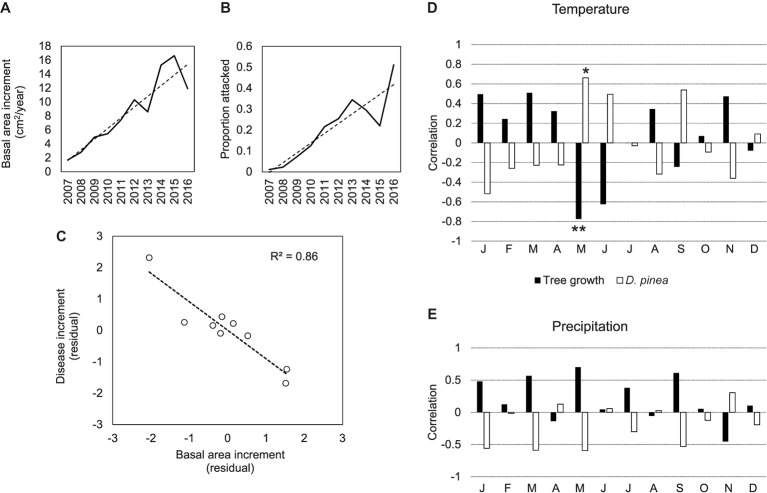
Temporal association between tree growth, *D. sapinea* epidemic, and weather conditions. **(A)** Tree radial growth based on basal area increments (BAI) and development of *D. sapinea* in the stand from 2007 to 2016 (*n* = 264 trees) **(B)**, correlation between standardized residuals from *D. sapinea* increments and BAI of 264 trees over 9 years **(C)**, correlation between average monthly temperatures **(D)** and precipitation **(E)** from 2007 to 2016 (*n* = 9 years) and standardized residuals from *D. sapinea* increments and BAI from 264 trees. Dashed lines in a, b, and c show adjusted linear regression. Significance levels in bar plots: **p < 0.01; *p < 0.05.

The number of putative *D. sapinea* attacks increased linearly over time (Figure 4A). In 2013 or 2016, the number of attacks was higher-than-expected, while in 2012, 2014, and 2015 the epidemic declined. In the years with largest attacks, trees grew less than expected (Figure 4B), and there was a strong negative correlation (*p* < 0.001) between disease increment and radial growth over time (Figure 4C). The same negative association between growth and *D. sapinea* was found in relation to weather conditions. Warm temperatures in May were associated with a lower tree growth (*p* = 0.004) and higher disease levels (*p* = 0.042) (Figure 4D). A nearly significant association was also found between low June temperature and growth (*p* = 0.054). No significant association between tree growth, disease, and precipitation was found (Figure 4E).

### Physiology of Attacked Trees

**Figure 5:**
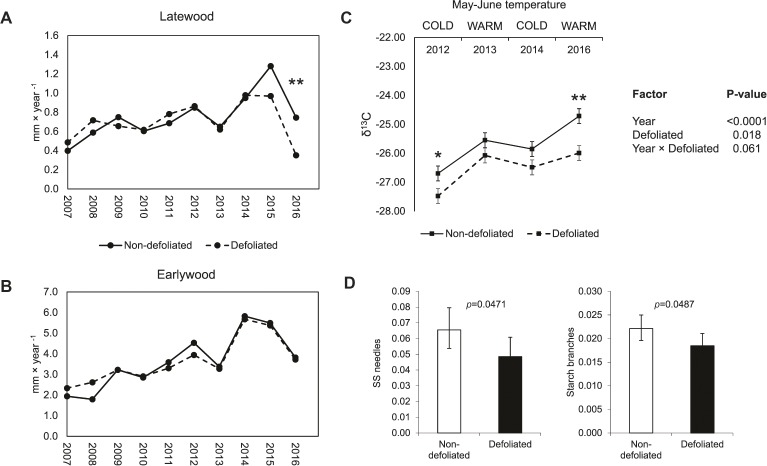
Comparative physiological performance between trees defoliated by *D. sapinea* (*n* = 12 trees) and non-defoliated trees (*n* = 12 trees). Latewood **(A)** and earlywood **(B)** widths in the period 2007–2016. **(C)** Carbon isotope ratio (δ^13^C) comparison between defoliated and non-defoliated trees across years when tree growth was high and spring conditions were cold (2012 to 2014) and years when spring was warm and growth was low (2013 and 2016). **(D)** Soluble sugar (SS) and starch concentration (% of dry weight) differences between defoliated and non-defoliated trees in needles and branch sapwood. Significant levels: **p < 0.01, *p < 0.05.

Comparing pairs of trees defoliated by *D. sapinea* and non-defoliated trees revealed that *D. sapinea* attacks affected mainly latewood production (Figure 5A). The year of the outbreak (2016) latewood was halved (0.74 vs. 0.35, *p* = 0.011), while no differences in terms of earlywood production were observed (Figure 5B). Heavily defoliated trees were characterized by displaying consistently lower δ^13^C values irrespective of whether the year had a warm or cold spring (Figure 5C). Defoliated trees had lower NSC concentrations in needles and branch sapwood during the winter after the outbreak (Figure 5D), but no differences were found in root NSCs.

### Spore Dynamics Within the Outbreak

**Figure 6:**
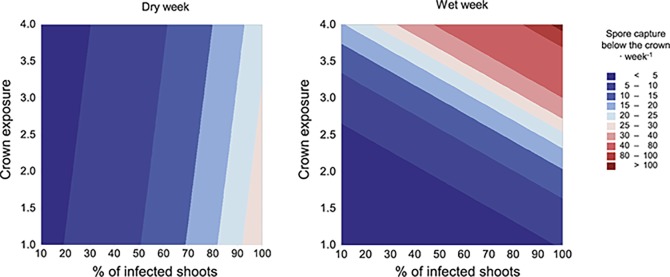
Weekly spore captures depending on the rain conditions, tree exposure, and percentage of infected shoots. Spores were trapped under each tree (*n* = 40 trees/traps during dry period, and *n* = 39 during wet period). Tree exposure ranges from 0 to 4 and measures how exposed to rain and wind was the crown of the tree: 0, tree surrounded by other trees; 4, tree growing in open space. No spore trap was placed under a tree with less than 10% defoliation or with a lower exposure than 1.

Spores collected during a wet period correlated with the amount of spores collected during dry conditions indicating a spatial consistency (*R* ^2^ = 0.59, *p* < 0.001). Spore captures tended to be larger in the southern part of the stand both under wet (*R* ^2^ = 0.25, *p* = 0.001) and dry weather conditions (*R* ^2^ = 0.59, *p* = 0.013). When considering the association between the amount of captured spores and the characteristics of the tree above the trap, we found a positive correlation with the percentage of dead shoots (*p* = 0.006) (Figure 6). During the wet week, also the exposure was associated with spore captures (*p* = 0.031 and *p* = 0.033, respectively for dead shoots and exposure), indicating that for a given level of damage, larger captures were obtained under crowns more exposed to wind and rain. Copy numbers obtained by qPCR on filter traps correlated significantly with spore captures on sticky slides (*R* ^2^ = 0.24, *p* < 0.001). However, the association tended to be lower (“week × spore number,” *p* = 0.060) during wet periods (4115 copies detected by qPCR/spore detected in the sticky trap) than during dry periods (1904 copies/spore).

## Discussion

Disease emergence has been traditionally associated with the lack of co-evolution between host and pathogen. However, the Swedish outbreak of *D. sapinea* was illustrative of how a pathogen can establish in a new area and emerge as a new endemic disease. Isolates found in the outbreak area were not genetically different from isolates obtained in previous surveys on asymptomatic forests in Sweden. The most dominant clone found in 2016 was already present in Sweden in 2013, initially discarding our first hypothesis of the outbreak being caused by a novel and more aggressive strain. The Swedish *D. sapinea* outbreak did not seem to fit either into the traditional picture of an introduced pathogen having problems to adapt to novel environmental conditions. Rather than causing intermittent boom-and-bust outbreaks, *D. sapinea* showed a good adaptation to a new environment, and its population displayed a steady increase over several years in the studied stand. Warm May and June temperatures apparently favored disease, which became more severe in the SW area of the stand, more exposed to sun and presumably warmer. Within that area, defoliation appeared on trees with a lower WUE, as indicated by their δ^13^C values. Symptomatic trees were the ones contributing more to the spread of the pathogen. Spores were captured during both wet and dry conditions; however, spore discharge seemed to be larger when affected crowns were more exposed to rain and wind. Our main finding is that northern conditions pose no apparent limitation for Diplodia tip blight. Nevertheless, further investigations should focus on understanding the origin of the inoculum of the outbreak.

We can only speculate with the origin of the inoculum of the outbreak area. One possible scenario is that *D. sapinea* was introduced in the stand with planting material bearing latent infections. We know that *D. sapinea* was present in Swedish nurseries during the 1960s (Molin et al., 1961) although the general prevalence in planting material is unknown. Work in Wisconsin (USA) has shown that incidence of *D. sapinea* could be as high as 30% of asymptomatic nursery stock (Stanosz et al., 2007) and that disease would only appear after seedlings would be subjected to water stress once out-planted. The absence of symptoms in the vicinity of the damaged stand fits well in the picture of a possible localized introduction from infected material in the stand of the outbreak; however, further investigations must be carried out to confirm that. Isolated trees showing severe symptoms were found in the immediacy of the outbreak, but those seem to fit better as part of the natural spread from the main stand, than as part of a widespread introduction. Recent surveys on spruce and pine seedlings in Sweden have not detected the pathogen (Menkis et al., 2016), so it seems that the current risk of *D. sapinea* being spread all over Sweden is rather low, although further surveys on the areas where seedlings from the same batch used in the outbreak area were planted should be undertaken.

*D. sapinea* seems to find highly favorable conditions to cause disease in Sweden, which is at odds with the absence of symptoms in the past. One possibility is that the disease has been misidentified, or perhaps confused with damages caused by *Melampsora* sp. Castagne, *Gremmeniella abietina* (Lagerb.) M. Morelet, or *Lophodermium seditiosum* Minter, Staley and Millar. In fact, and based on the reconstruction of damages, severity should have been high in previous years, and no one reported them. Overlooking severe damages in the forest may be not so unlikely after all. The outbreak in 2016 affected trees that were next to the highway, and no one noticed the damages even severity was ca. 90%. Another possibility is that *D. sapinea* outbreak in 2016 responded to very special conditions that precluded an explosion of damages. However, 2016 did not seem to be extreme regarding any of the weather variables considered. The possibility of a local haze storm or other phenomena cannot be discarded. However, the reconstruction of the epidemic based on tree bifurcations seems to bring the origin of the outbreak way back in time.

It is unclear which could be the underlying mechanism behind the association between *D. sapinea* attacks and warm conditions during May and June. In May and June, shoots start to develop, but completion ends more towards end of June, when correlation was no longer significant. Warm temperatures may enable the pathogen to develop endophytically in the bud immediately before sprouting (Brookhouser and Peterson, 1970). Alternatively, warm temperatures could increase drought stress in the new shoots, a condition that has been found associated with *D. sapinea* damages in ours and in previous reports (Bachi and Peterson, 1985; Stanosz et al., 2001). We observed that warm conditions in spring seemed to be detrimental for tree growth, pointing to the fact that high temperatures during shoot elongation may pose some stress to the tree. The importance of thermal conditions of May and June could also be associated with day length and photosynthetic activity. During summer, in northern locations, trees may be active for longer hours every day than in southern locations, extending the period of time when shoots may become susceptible due to lower water potentials caused by photosynthesis. As an example, the nearby city of Stockholm has an average of 18 hours of daylight on 1^st^ June. A lack of lignification during early shoot development could also be associated with a higher susceptibility (Jalkanen and Kurkela, 1984; Petäistö and Repo, 1988; Petäistö, 1999).

The clonal structure found across Europe supports the idea of a predominantly asexual reproduction in *D. sapinea* in the continent. The low variability observed among our populations may be due to the low resolution of the markers. However, genotypic diversity in our study was similar to that found in other studies in South Africa (Burgess et al., 2001; Bihon et al., 2011). That and the fact that markers were able to detect a diversity hotspot in Turkey seems to reject resolution limitations. A certain level of adaptation cannot be fully discarded since there seems to be a geographical pattern across Europe, as observed in more localized studies (Luchi et al., 2014). However, without phenotypic data on the isolates, it is not possible to link the genetic structure with some sort of adaptation to northern latitudes, as done for other pathogens such as *Heterobasidion parviporum* Niemelä and Korhonen (Müller et al., 2015). Further investigations on survival and pathogenicity of northern *D. sapinea* isolates should be carried out.

A more liberal use of water seemed to a key phenotype associated with susceptibility. Even though there was some variation in terms of δ^13^C across years, no association with high or low temperatures was found, and rather it seemed that the differences in terms of WUE were consistent between symptomatic and asymptomatic trees irrespective of growing under favorable/unfavorable conditions. Previous studies have brought up the role of tree phenotype increasing susceptibility (Witzell and Karlman, 2000; Oliva et al., 2014, 2016), which, in northern conditions, seems to be relating fast growth with disease (Stenlid and Oliva, 2016). This issue is particularly important in northern areas where forest regeneration is mostly done by planting and where growth expectations in high site index areas may carry a higher susceptibility. In Scandinavia, Scots pine and Norway spruce may be alternatively planted in the same stand (as in the case of the outbreak area), and therefore, provenances carrying more resistant phenotypes could be used as a prevention strategy in areas with high inoculum pressure.

Spore captures showed that spread is possible under wet or dry conditions, in contrast with previous studies where a more seasonal dispersal was found (Brookhouser and Peterson, 1971; Swart et al., 1987). We only sampled two weeks in autumn; thus, further experiments should be carried out to find whether these findings hold during summer or spring. Nevertheless, our results pointed to a different spore dynamic during rainy and dry weather. In both, captures were larger under more damaged trees. Spore deposition within the same tree may be an important component of the epidemic, as shown by the fact that highly damaged trees were also those with more attacks in the past. However under rainy conditions, crown exposure also favored dispersal. One possibility is that under an open crown the spore trap captured not only spores from the immediate tree but also from the surroundings. Also, a more open crown may facilitate a better wetting of the crown and a higher number of pycnidia being hydrated and releasing spores. Combining spore trapping on filter papers with qPCR seemed to be a powerful tool to monitor *D. sapinea*, although the lower efficiency under rainy conditions needs to be considered.

*D. sapinea* proved to be a damaging pathogen in economic terms, not so much because of its impact on growth, as losses were mainly seen on the latewood production (representing a small fraction of the ring), but because of its capacity to kill the leader shoot(s), disrupt the shape of the growing crown, and decrease the quality of the stem. Impact on NSC reserves was localized in proximal tissues to the attack area (needles and shoots), but no overall effect was detected in the root system where a large amount of NSCs are stored during winter. *D. sapinea* seemed to be able to survive well under nearly boreal conditions; therefore, disease management should focus on reducing inoculum. Inoculum build-up seems to be favored by warm conditions in spring, which may have implications under future climate projections. In Sweden, spring temperatures have shown a steady increase over the last decades, while summer, autumn, and winter temperatures seem more stable.^2^ Seedlings and also seeds should be carefully inspected as *D. sapinea* may be present in a latent stage in asymptomatic tissues.

## Author Contributions

JO discovered the outbreak, coordinated the project, performed field work, analyzed the data, and drafted a first version of the manuscript. LB performed field measures, isolations, and molecular work, including population structure analyses. JC and ÁS-M did the dendrochronological, NSC, and isotopic measures. KA, CC, RD, NL, DM, AL, RD, and ŞÖ sampled *D. sapinea* in their respective countries and provided isolates or DNA. JS contributed designing the experiment. JO, JS, and RD funded the project. All authors contributed to the manuscript writing.

### Conflict of Interest Statement

The authors declare that the research was conducted in the absence of any commercial or financial relationships that could be construed as a potential conflict of interest.

## Footnotes

^1^ luftweb.smhi.se

^2^ www.smhi.se

## References

[ref1] Adamson K. Klavina D. Drenkhan R. Gaitnieks T. Hanso M. (2015). *Diplodia sapinea* is colonizing the native scots pine (*Pinus sylvestris*) in the northern baltics. Eur. J. Plant Pathol. 143, 343–350. 10.1007/s10658-015-0686-8

[ref2] Bachi P. R. Peterson J. L. (1985). Enhancement of *Sphaeropsis sapinea* stem invasion of pines by water deficits. Plant Dis. 69, 798–799.

[ref3] Bihon W. Burgess T. Slippers B. Wingfield M. J. Wingfield B. D. (2011). Distribution of *Diplodia pinea* and its genotypic diversity within asymptomatic *Pinus patula* trees. Australas. Plant Pathol. 40, 540–548. 10.1007/s13313-011-0060-z

[ref4] Bosso L. Luchi N. Maresi G. Cristinzio G. Smeraldo S. Russo D. (2017). Predicting current and future disease outbreaks of *Diplodia sapinea* shoot blight in Italy: species distribution models as a tool for forest management planning. For. Ecol. Manag. 400, 655–664. 10.1016/j.foreco.2017.06.044

[ref5] Brookhouser L. W. Peterson G. W. (1970). Infection of Austrian, Scots, and ponderosa pines by *Diplodia pinea*. Phytopathology 61, 409–414. 10.1094/Phyto-61-409

[ref6] Burgess T. Wingfield M. J. Wingfield B. W. Kay S. J. Farrell R. L. Hofstra D. (2001). Simple sequence repeat markers distinguish among morphotypes of *Sphaeropsis sapinea*. Appl. Environ. Microbiol. 67, 354–362. 10.1128/AEM.67.1.354-362.2001, PMID: 11133466 PMC92584

[ref7] Burgess T. I. Wingfield M. J. Wingfield B. D. (2004). Global distribution of *Diplodia pinea* genotypes revealed using simple sequence repeat (SSR) markers. Australas. Plant Pathol. 33, 513–519. 10.1071/AP04067

[ref8] Buysse J. A. N. Merckx R. (1993). An improved colorimetric method to quantify sugar content of plant tissue. J. Exp. Bot. 44, 1627–1629. 10.1093/jxb/44.10.1627

[ref9] Cheng-guo W. Blanchette R. Jackson W. Palmer M. (1985). Differences in conidial morphology among isolates of *Sphaeropsis sapinea*. Plant Dis. 69, 838–841. 10.1094/pd-69-838

[ref104] Chou C. K. S. (1987). Crown wilt of *Pinus radiata* associated with *Diplodia pinea* infection of woody stems. Eur. J. Forest Pathol. 17, 398–411.

[ref10] Fabre B. Piou D. Desprez-Loustau M.-L. Marçais B. (2011). Can the emergence of pine *Diplodia* shoot blight in France be explained by changes in pathogen pressure linked to climate change? Glob. Chang. Biol. 17, 3218–3227. 10.1111/j.1365-2486.2011.02428.x

[ref11] Françoise D. C. Hugo D. Xavier C. Olivier F. Sylvain D. Marie-Laure D. L. (2015). Escape of spring frost and disease through phenological variations in oak populations along elevation gradients. J. Ecol. 103, 1044–1056. 10.1111/1365-2745.12403

[ref12] Gardes M. Bruns T. D. (1993). ITS primers with enhanced specificity for basidiomycetes–application to the identification of mycorrhizae and rusts. Mol. Ecol. 2, 113–118. 10.1111/j.1365-294X.1993.tb00005.x, PMID: 8180733

[ref13] Hanso M. Drenkhan R. (2009). *Diplodia pinea* is a new pathogen on Austrian pine (*Pinus nigra*) in Estonia. Plant Pathol. 58, 797–797. 10.1111/j.1365-3059.2009.02082.x

[ref14] Hanso M., Drenkhan R. (2013). Simple visualization of climate change for improving the public perception in forest pathology. For. Stud. 58, 37–45. 10.2478/fsmu-2013-0004

[ref15] Holmes R. L. (1983). Computer-assisted quality control in tree-ring dating and measurement. Tree-Ring Bull. 43, 68–78.

[ref16] Jalkanen R. Kurkela T. (1984). Damage and early height growth losses caused by *Melampsora pinitorqua* on Scots pine. Folia For. 587, 1–15.

[ref17] Jung T. Orlikowski L. Henricot B. Abad-Campos P. Aday A. G. Aguín Casal O. (2016). Widespread *Phytophthora* infestations in European nurseries put forest, semi-natural and horticultural ecosystems at high risk of *Phytophthora* diseases. For. Pathol. 43, 134–163. 10.1111/efp.12239

[ref18] Kamvar Z. N. Tabima J. F. Grunwald N. J. (2014). Poppr: an R package for genetic analysis of populations with clonal, partially clonal, and/or sexual reproduction. Peer. J. 2:e281. 10.7717/peerj.281, PMID: 24688859 PMC3961149

[ref19] La Porta N. Capretti P. Thomsen I. M. Kasanen R. Hietala A. M. Von Weissenberg K. (2008). Forest pathogens with higher damage potential due to climate change in Europe. Can. J. Plant Pathol. 30, 177–195. 10.1080/07060661.2008.10540534

[ref20] Liebhold A. M. Brockerhoff E. G. Garrett L. J. Parke J. L. Britton K. O. (2012). Live plant imports: the major pathway for forest insect and pathogen invasions of the US. Front. Ecol. Environ. 10, 135–143. 10.1890/110198

[ref21] Luchi N. Capretti P. Bonello P. (2007). Production of *Diplodia scrobiculata* and *Diplodia pinea* pycnidia on ground Austrian pine needle agar medium. Phytopathol. Mediterr. 46, 230–235. 10.14601/Phytopathol_Mediterr-2157

[ref22] Luchi N. Capretti P. Surico G. Orlando C. Pazzagli M. Pinzani P. (2005). A real-time quantitative PCR assay for the detection of *Sphaeropsis sapinea* from inoculated *Pinus nigra* shoots. J. Phytopathol. 153, 37–42. 10.1111/j.1439-0434.2004.00924.x

[ref23] Luchi N. Oliveira Longa C. M. Danti R. Capretti P. Maresi G. (2014). *Diplodia sapinea*: the main fungal species involved in the colonization of pine shoots in Italy. For. Pathol. 44, 372–381. 10.1111/efp.12109

[ref24] Menkis A. Burokienė D. Stenlid J. Stenström E. (2016). High-throughput sequencing shows high fungal diversity and community segregation in the rhizospheres of container-grown conifer seedlings. Forests 7:44. 10.3390/f7020044

[ref25] Millberg H. Hopkins A. J. M. Boberg J. Davydenko K. Stenlid J. (2016). Disease development of *Dothistroma* needle blight in seedlings of *Pinus sylvestris* and *Pinus contorta* under nordic conditions. For. Pathol. 46, 515–521. 10.1111/efp.12242

[ref72] Molin N. Persson M. Persson S. (1961). Root parasites on forest tree seedlings. Some exploratory tests of the resistance of germinant seedlings and the virulence of some potential parasites. Meddelande från Statens Skogforskningsinstitut 49, 1–16.

[ref26] Müller M. M. Hamberg L. Kuuskeri J. LaPorta N. Pavlov I. Korhonen K. (2015). Respiration rate determinations suggest *Heterobasidion parviporum* subpopulations have potential to adapt to global warming. For. Pathol. 45, 515–524. 10.1111/efp.12203

[ref500] Müller M. M. Hantula J. Wingfield M. Drenkhan R. (2018). *Diplodia sapinea* found on Scots pine in Finland. *For. Pathol*. 2018:e12483. 10.1111/efp.12483

[ref27] Oliva J. Boberg J. Stenlid J. (2013). First report of *Sphaeropsis sapinea* on Scots pine (*Pinus sylvestris*) and Austrian pine (*P. nigra*) in Sweden. New Dis. Rep. 27:23. 10.5197/j.2044-0588.2013.027.023

[ref28] Oliva J. Stenlid J. Grönkvist-Wichmann L. Wahlström K. Jonsson M. Drobyshev I. (2016). Pathogen-induced defoliation of *Pinus sylvestris* leads to tree decline and death from secondary biotic factors. For. Ecol. Manag. 379, 273–280. 10.1016/j.foreco.2016.08.011

[ref29] Oliva J. Stenlid J. Martínez-Vilalta J. (2014). The effect of fungal pathogens on the water and carbon economy of trees: implications for drought-induced mortality. New Phytol. 203, 1028–1035. 10.1111/nph.12857, PMID: 24824859

[ref30] Palacio S. Maestro M. Montserrat-Martí G. (2007). Seasonal dynamics of non-structural carbohydrates in two species of mediterranean sub-shrubs with different leaf phenology. Environ. Exp. Bot. 59, 34–42. 10.1016/j.envexpbot.2005.10.003

[ref31] Palmer M. Stewart E. Wingfield M. (1987). Variation among isolates of *Sphaeropsis sapinea* in North Central United States. Phytopathology 77, 944–948. 10.1094/Phyto-77-944

[ref32] Petäistö R. L. (1999). Growth phase of bare-root Scots pine seedlings and their susceptibility to *Gremmeniella abietina*. Silva Fenn. 33, 179–185. 10.14214/sf.655

[ref33] Petäistö R. L. Repo T. (1988). Stress combinations and the susceptibility of *Scots pine* to *Ascocalyx abietina*. Mitt. Forstl. Bundes-Vers.anst. Wien 162, 103–118.

[ref34] Phillips A. J. L. Alves A. Abdollahzadeh J. Slippers B. Wingfield M. J. Groenewald J. Z. (2013). The botryosphaeriaceae: genera and species known from culture. Stud. Mycol. 76, 51–167. 10.3114/sim0021, PMID: 24302790 PMC3825232

[ref35] Redondo M. A. Boberg J. Olsson C. H. Oliva J. (2015). Winter conditions correlate with *Phytophthora alni* subspecies distribution in Southern Sweden. Phytopathology 105, 1191–1197. 10.1094/PHYTO-01-15-0020-R, PMID: 25822186

[ref36] Redondo M. A. Boberg J. Stenlid J. Oliva J. (2018a) Contrasting distribution patterns between aquatic and terrestrial *Phytophthora* species along a climatic gradient are linked to functional traits. ISME J. 12, 2967–2880. 10.1038/s41396-018-0229-3 30072746 PMC6246556

[ref37] Redondo M. Á. Boberg J. Stenlid J., Oliva J. (2018b). Functional traits associated with the establishment of introduced *Phytophthora* spp. in Swedish forests. J. Appl. Ecol. 55, 1538–1552.

[ref38] Smith D. R. Stanosz G. R. (2006). A species-specific PCR assay for detection of *Diplodia pinea* and *D. scrobiculata* in dead red and jack pines with collar rot symptoms. Plant Dis. 90, 307–313. 10.1094/PD-90-0307 30786554

[ref39] Stanosz G. R. Blodgett J. T. Smith D. R. Kruger E. L. (2001). Water stress and *Sphaeropsis sapinea* as a latent pathogen of red pine seedlings. New Phytol. 149, 531–538. 10.1046/j.1469-8137.2001.00052.x 33873334

[ref40] Stanosz G. R. Smith D. R., Leisso R. (2007). Diplodia shoot blight and asymptomatic persistence of *Diplodia pinea* on or in stems of jack pine nursery seedlings. For. Pathol. 37, 145–154. 10.1111/j.1439-0329.2007.00487.x

[ref103] Stanosz G. R. Smith D. R. Guthmiller M. A. Stanosz J. C. (1997). Persistence of *Sphaeropsis sapinea* on or in asymptomaticsshoots of red and Jack pines. Mycologia 89, 525–530.

[ref41] Stenlid J. Oliva J. (2016). Phenotypic interactions between tree hosts and invasive forest pathogens in the light of globalization and climate change. Philos. T. Roy. Soc. B. 371:20150455. 10.1098/rstb.2015.0455 PMC5095534 28080981

[ref42] Sturrock R. N. Frankel S. J. Brown A. V. Hennon P. E. Kliejunas J. T. Lewis K. J. (2011). Climate change and forest diseases. Plant Pathol. 60, 133–149. 10.1111/j.1365-3059.2010.02406.x

[ref43] Swart W. J. Wingfiled M. J. Knox-Davies P. S. (1987). Conidial dispersion of *Sphaeropsis sapinea* in three climatic regions of South Africa. Plant Dis. 71, 1038–1040. 10.1094/PD-71-1038

[ref44] Wang H. Qi M. Cutler A. J. (1993). A simple method of preparing plant samples for PCR. Nucleic Acids Res. 21, 4153–4154. 10.1093/nar/21.17.4153, PMID: 8371994 PMC310032

[ref45] Witzell J. Karlman M. (2000). Importance of site type and tree species on disease incidence of *Gremmeniella abietina* in areas with a harsh climate in Northern Sweden. Scand. J Forest Res. 15, 202–209. 10.1080/028275800750015019

[ref46] Woods A. Coates K. D. Hamann A. (2005). Is an unprecedented dothistroma needle blight epidemic related to climate change? Bioscience 55, 761–769. 10.1641/0006-3568(2005)055[0761:IAUDNB]2.0.CO;2

[ref102] Zwolinski J. B. Swart W. J. Wingfield M. J. (1990). Economic impact of a post-hail outbreak of dieback induced by *Sphaeropsis sapinea*. Eur. J. Forest Pathol. 20, 405–411.

